# Quantifying the Economic and Cultural Biases of Social Media through Trending Topics

**DOI:** 10.1371/journal.pone.0134407

**Published:** 2015-07-31

**Authors:** Juan Miguel Carrascosa, Ruben Cuevas, Roberto Gonzalez, Arturo Azcorra, David Garcia

**Affiliations:** 1 Universidad Carlos III de Madrid, Leganés, Madrid, Spain; 2 NEC Laboratories, Heildelberg, Baden-Württemberg, Germany; 3 Institute IMDEA Networks, Leganés, Madrid, Spain; 4 ETH Zurich, Zurich, Switzerland; University of Zaragoza, SPAIN

## Abstract

Online social media has recently irrupted as the last major venue for the propagation of news and cultural content, competing with traditional mass media and allowing citizens to access new sources of information. In this paper, we study collectively filtered news and popular content in Twitter, known as Trending Topics (TTs), to quantify the extent to which they show similar biases known for mass media. We use two datasets collected in 2013 and 2014, including more than 300.000 TTs from 62 countries. The existing patterns of leader-follower relationships among countries reveal systemic biases known for mass media: Countries concentrate their attention to small groups of other countries, generating a pattern of centralization in which TTs follow the gradient of wealth across countries. At the same time, we find subjective biases within language communities linked to the cultural similarity of countries, in which countries with closer cultures and shared languages tend to follow each other’s TTs. Moreover, using a novel methodology based on the Google News service, we study the influence of mass media in TTs for four countries. We find that roughly half of the TTs in Twitter overlap with news reported by mass media, and that the rest of TTs are more likely to spread internationally within Twitter. Our results confirm that online social media have the power to independently spread content beyond mass media, but at the same time social media content follows economic incentives and is subject to cultural factors and language barriers.

## Introduction

Since the existence of online social media, citizens around the world use it to communicate beyond mass media blackouts. For example, IRC channels served as a way for individuals to report news in 1991 during the media blocks in the Soviet Union coup de etat and in the Gulf War [1]. The growth of social media use in developed societies allowed individuals to take one step further, organizing actions and spreading relevant information around their environment. One example of such emergence of coordination away from mass media are the actions of the *Anonymous* group [2], which in 2008 organized demonstrations and produced reports against the Church of Scientology. More recently, the widespread adoption of social media around the world has triggered events that were reported by individuals beyond media blockages, including actions of social movements like the Spanish *Indignados* [3], the Gezi protests in Turkey [4], and the revolutions during the Arab Spring [5].

While social media are clearly relevant for news and culture, there are still many open questions about the potential, role, limitations, and biases of online social media. Social media overcome some limitations of traditional mass media that are commonly attributed as sources of biases. First, the cost to set up an information channel in social media is negligible, allowing individual users to become information channels themselves. This overcomes the ownership barrier of traditional media [6] and potentially weaken biases related to information centralization. Second, social media have the potential of a very broad and deep coverage of all kinds of content, allowing any information to be found, reported, and eventually attract collective attention. But such potential might not necessarily be realized, in particular when information overloads and misinformation spread due to the communication brevity and informality of many social media platforms. In addition, social media are not free of the influence of other biases that can limit their transparency and coverage. For example, a major part of the funding in social media comes from advertising strategies, in which the product is the attention of users and not the reported content [7]. Furthermore, social media are not isolated communities, and traditional mass media biases can potentially resonate in each social medium. A recent example of selective reporting in both mass and social media is the reaction to the Charlie Hebdo shootings in January 2015, which received an extremely large attention share in comparison to similar attacks against freedom of speech in former Yugoslavia or the Middle East [8].

The selection and content of centralized media can be affected by *subjective* and/or *systemic* biases. Subjective biases operate at the level of the individual information of the reporter, during the evaluation of the informativeness in the context of current events [9]. Since online media content is collectively curated by large groups of Internet users, other subjective biases can emerge from shared values [10], information overloads [11], and cultural preferences [12]. Systemic biases operate at a mesoscopic level, creating patterns that cannot be observed at the level of a news piece or a journalist, but can be observed at larger scales when sufficient content is analyzed [6]. In the context of international news, there is no evidence that can attribute these biases to supranational power structures [13], but incentive mechanisms can bias mass media through economic and social forces [6, 14]. Examples of empirically tested presence of these systemic biases relate them to the increase of reporting with Gross Domestic Product (GDP) of the country where news originate [15], and its decrease with geographical distance [16] and political stability [17]. This theory is still applicable to online social media as part of a larger medium that also includes traditional mass media.

With respect to social media, previous works focused either on individual biases in news sharing [18–20], or on social and geographical factors or content sharing relevant to viral marketing and information technologies [21–23]. Within this context of individual behavior, the concept of algorithmic biases in personalization [24] add a complementary view to our collective analysis on systemic and subjective biases. Algorithmic biases are often related to the filter bubble [25], a phenomenon that creates echo chambers [26] that build on already existing individual confirmatory biases [27]. While such concept can be linked to polarization phenomena [28], we focus on testing the economic and cultural factors hypothesized by the theories of media biases. Our approach covers a wider spectrum of online social media, in which not only breaking news play a role, but also popular content like gossip, cultural trends, and fashion define the information that is considered relevant and consumed across societies.

In a formal sense, we analyze collective information filters, which contain selected information of temporal relevance that gains special visibility in a centralized communication channel [29]. While the datasources for mass media are defined by newspapers, magazines, and television programs, such content in online social media appears in link sharing communities, like Reddit [30], and aggregation mechanisms in microblogging platforms. In this article, we focus on Twitter Trending Topics (TTs), which serve as a global filtering mechanism for Twitter users to select which content is relevant and when. This way, TTs serve as a centralized channel of communication from many to many, serving the purpose of central media within Twitter due to the public interest, temporal component, and global reach of TTs through the Twitter interface. We present our study of media biases in TTs in two large-scale datasets from 2013 and 2014, detecting temporal patterns of TT appearance that reveal the network of media influence across countries. Based on that network, we perform a confirmatory analysis to test systemic and subjective biases in social media, and an exploratory analysis on the relation between TTs and mass media.

## Materials and Methods

### Data on Trending Topics

We gathered a dataset of Local TTs in Twitter: Phrases or words that appear in Twitter at a much higher frequency than the rest in a certain country during a short period of time. Local TTs serve as crowdsourced aggregators of recent topics of high relevance and popularity, filtering the activity of a country through a centralized channel of temporally relevant information. Among the information provided by its REST API, Twitter provides the list of 10 Local TTs at query time in a given location (e.g., a country). We retrieved the list of TTs at all the available countries in an automated fashion, gathering the list of Local TTs for each country every 5 minutes, which is the interval used by Twitter to update the list of TTs [31].

We executed our retrieval software in two study periods: February 20th to May 20th, 2013 and April 14th to July 4th, 2014. During each study period, we collected information on all available countries with Local TTs provided by the API, which amounts to 35 countries (identified by their ISO-3166 codes) in the first period and 62 countries in the second one (including the 35 previous ones). The first study period produced a dataset of more than 112.000 Local TTs and the second period a set of more than 188.000 TTs. We refer to these datasets as TT-2013 and TT-2014, respectively, and are publicly available at [32]. Finally, it is worth noting that our data collection methodology is compliant with Twitter’s terms of Use and Service [33]. Moreover, since we are able to collect all TTs for every available country at a rate equal to the TTs’ update interval configured by Twitter, we conclude that there is no bias in the obtained results due to the data collection methodology.

### Data on demographic, economic, and cultural factors

For each of the 62 countries, we collect a dataset to quantify variables that are subject to induced biases in the appearance of TTs. We measure the wealth of a country through its GDP using purchasing power parity rates as reported by the World Bank statistics of 2010 (data on purchasing power parity from Argentina during the study period is not available in the World Bank). We also obtain the most recent directed migration statistics, from 2000, between each pair of countries from the World Bank database. For each country, we set its timezone as the UTC offset in winter time of the largest city as reported by Wikipedia. Furthermore, we also extract from Wikipedia a list of official and national languages in each country.

We quantify the culture of each country *c* using the four principal cultural dimensions of Hofstede’s model [10]: power distance *p*_*c*_, individualism *i*_*c*_, masculinity *m*_*c*_, and uncertainty avoidance *u*_*c*_. Our intention applying Hofstede’s dimensions is not to explore the role of any particular aspect of culture, but to quantify the distance between cultures as an aggregate of the differences in the shared values of two societies. While some countries are not included in Hofstede’s dataset, 31 countries from TT-2013 and 43 countries from TT-2014 are present.

### Detecting mass media influence in Twitter

We developed a method to determine if a TT appearing in country *c* at a given date *d* is related to content reported in traditional mass media (e.g., a newspaper). If a TT is related to at least one mass media item in the country *c* during a time window of *d*±*N* days, we consider the TT as *External*, and *Internal* to Twitter if it did not appear in mass media. We use Google News to detect if a TT is reported in the mass media of the country, querying for certain terms related to the TT for the interval of *N* days before and after the TT emerged. We apply this method to the TTs of four countries (US, ES, CA and GB) collected over a period of time of 1 month between April and May, 2014. Furthermore, we consider a time window of 5 days (*N* = 2) around the appearance of the TT. More technical details on this method and its validation are provided in S1 Text.

## Results

### Leader-Follower structures

The temporal sequence of appearance of Local TTs allows us to analyze the structure of leader-follower relationships among countries in Twitter. This type of relationship constitutes a media bias in which the temporal patterns of news and popular content are a manifestation of an alignment of incentives, rather than a causal relationship. Herman and Chomsky detail this kind of bias in their Propaganda model [6], operationalizing it as a *sourcing filter* in which some sources are overlooked, distorting the information presented to the public. Thus, leader-follower relationships can appear without a hidden power that manipulates media outlets in different countries; they can be the product of a set of shared interests that create ordered patterns where content originates and where it is consumed afterwards.

We detect leader-follower relationships among countries through the ordering of the appearance of Local TTs. If such relationship exists, there will be a tendency for Local TTs to appear in the *leader* country before they emerge in the *follower* country. Thus, we take into account in our analysis the events in which a Local TT appears in country *C*_*i*_ at time *t*_*i*_, and later in country *C*_*j*_ at time *t*_*j*_ > *t*_*i*_. To test if these temporal sequences are not the product of independent events unrelated to leader-follower relationships, we apply the model of priority processes and bursty patterns in queue theory [34, 35]. If Local TTs appear in pairs of countries by chance, as the result of independent phenomena, the time intervals between the appearances Δ*t* will follow an exponential distribution *P*(Δ*t*) ∼ *λe* ^−*λ*Δ*t*^ as the result of decoupled Poisson processes. On the other hand, if the appearances of Local TTs are correlated in time, the time sequence will make the distribution of Δ*t* to follow a power-law *P*(Δ*t*) ∼ Δ*t* ^−*α*^ [35]. The exponent *α* of this power-law is characteristic of which kind of dynamics produce the correlation. The case of *α* = 2.5 corresponds to exogenously triggered dynamics in which external events produce the TTs [36]; *α* = 1.5 indicates an endogenous process with leader-follower relationships with infinite queues; and *α* = 1 top the same process but with finite queues [35]. This kind of temporal patterns are known to appear in communication processes, including the correspondence of Einstein and Darwin [34], e-mail communication [35], chatroom interaction [37], and Twitter dialogues [38].

**Fig 1:**
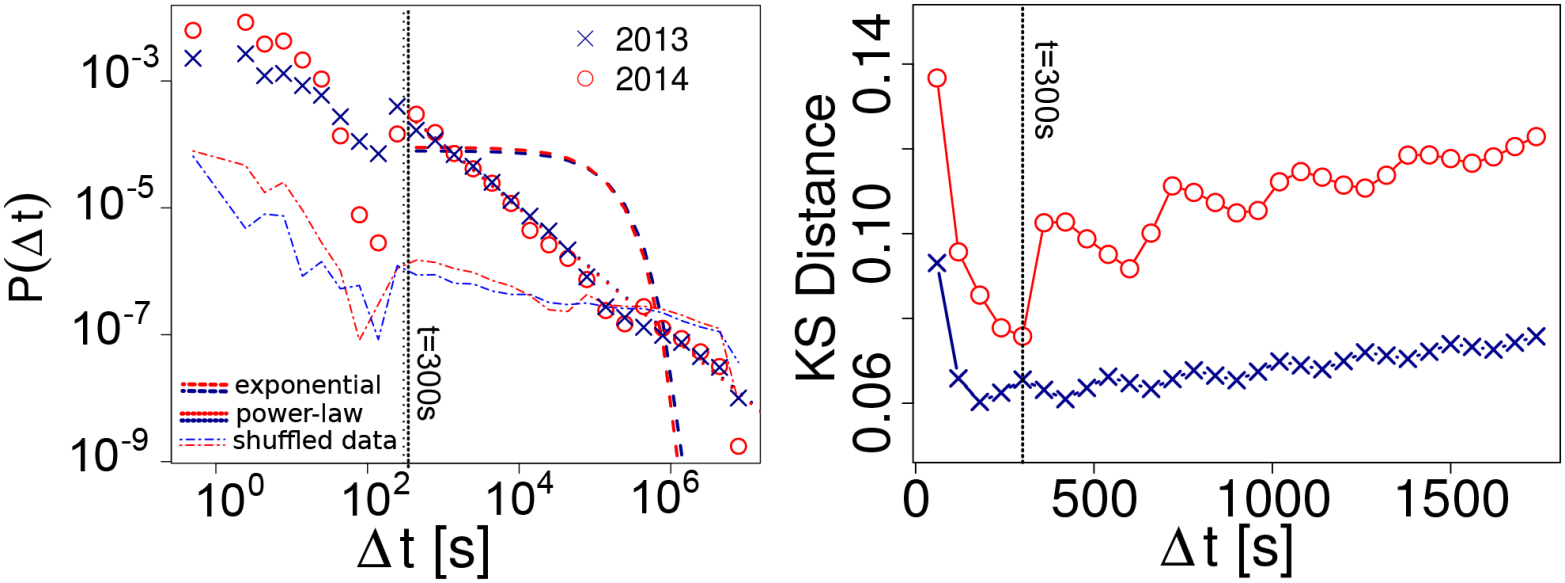
Analysis of time intervals between the appearance of TT. Left: distribution of time intervals, dashed lines show power-law, exponential fits, and distributions for shuffled datasets. Right: KS Distance between the empirical distribution and a power-law fit for ranging values of the minimum Δ*t* of the fit.

The left panel of Fig 1 shows the distributions of times between the appearance of TTs in different countries *P*(Δ*t*), for both TT-2013 and TT-2014 datasets. To test the alternative hypotheses of the existence or nonexistence of correlations between TTs appearances, we fit power-law and exponential distributions through maximum likelihood and the Kolmogorov-Smirnov criterion [39] (more details in the S1 Text). The resulting theoretical distributions are plotted in Fig 1, suggesting that a power-law fit is better than the exponential alternative. Log likelihood ratio tests [40] between the exponential and power-law models give significant estimates of 198.22 (TT-2013) and 293.03 (TT-2014), providing very strong support to reject the independent events hypothesis, in favor of the hypothesis that the appearance of TTs follows a correlated process. We further test this conclusion by shuffling the TTs coocurrence data, permuting the timestamps of appearance of each TT in leader and follower countries. This way, we randomize the pairs, but keep the empirical properties of the distributions of TT appearance in each country. The probability of finding a short interval between the appearance of TTs in this ancillary dataset are much lower than in the empirical distribution, as shown in the left panel of Fig 1. Furthermore power-law fits fail in comparison to exponential fits, allowing us to conclude that the empirical shape of *P*(Δ*t*) is far from random.

The exponent of the fits are *α*_*TT*−2013_ = 1.00005±10^−6^ and *α*_*TT*−2014_ = 1.004±10^−5^, indicating that the process of TTs appearance is not due to exogenous factors but depends on leader-follower relationships with finite queues [35]. This also indicates that countries have strong limitations in their capacity to adopt TTs in comparison to how many are generated in the rest of the world. However, note that these correlations are not observable at all timescales, as the power-law distributions are fit above a minimum value of Δ*t*. The right panel of Fig 1 shows the Kolmogorov-Smirnov estimate (KS) for a set of minimum values, revealing that the minimum for TT-2013 and TT-2014 are 3 and 5 minutes, respectively. This means that correlations at a timescale smaller than 3 minutes cannot be observed in any of our datasets, and that between 3 and 5 minutes the results are inconsistent. Thus, we take 5 minutes as a minimum criterion to deduce the manifestation of a leader-follower relation. This coincides with our sampling frequency, allowing us to remove from the data those cooccurrences for which we do not have sufficient evidence to consider them product of a leader-follower process.

**Fig 2:**
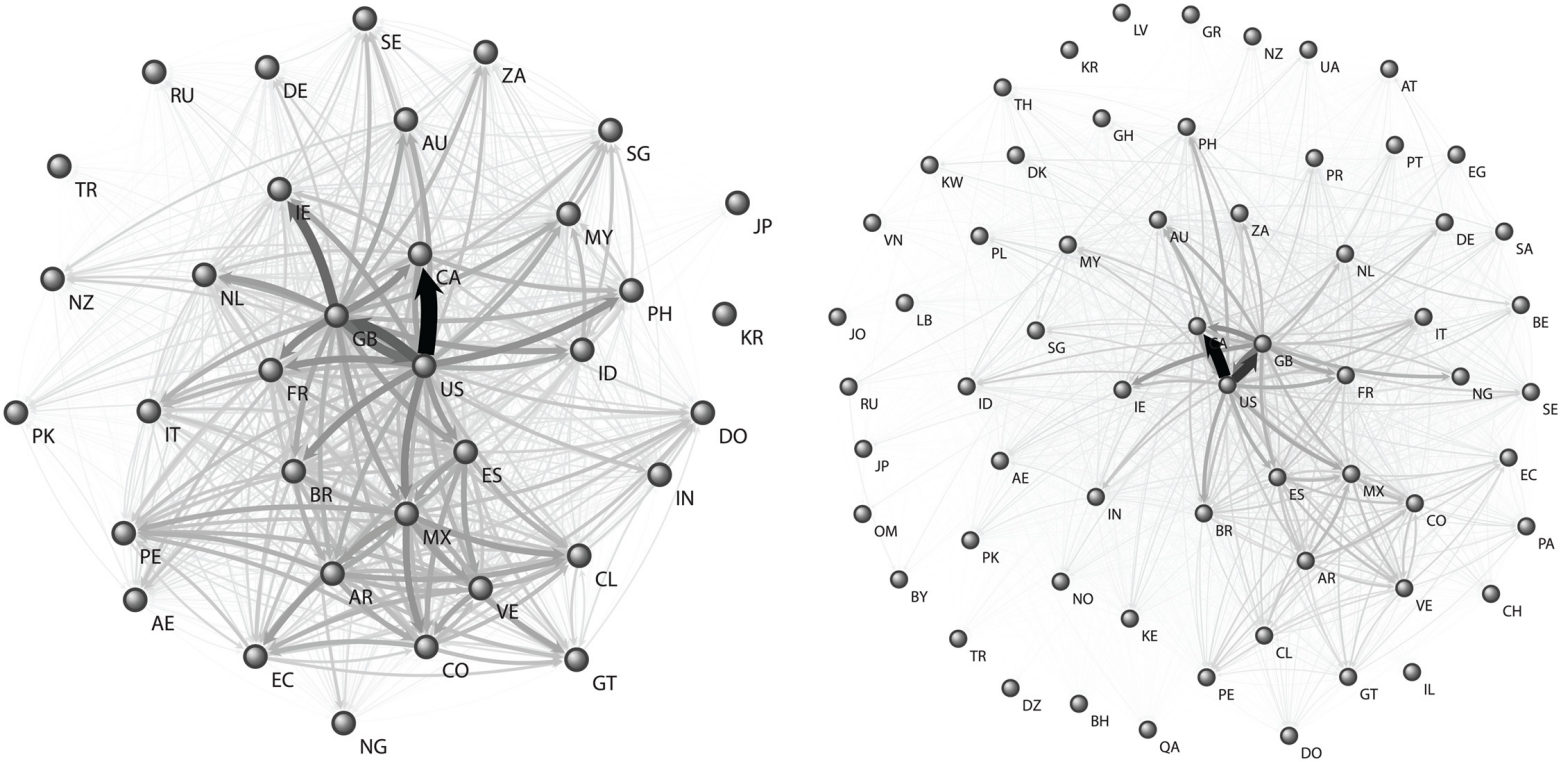
Visual Representation of the International structure of TTs. TT-2013 is shown on the left and TT-2014 on the right. The size and darkness of the links are proportional to their weights.

We quantify the tendency for country *C*_*f*_ to follow country *C*_*l*_ through the amount of TTs that appeared in *C*_*f*_ at least 5 minutes after they appeared in *C*_*l*_. When applied to every pair of countries in our dataset, these counts define a weighted, directed network in which nodes are countries and links have weights corresponding to the number of TTs that appeared in the leader-follower relationship. We refer to this graph, shown in Fig 2 for TT-2013 and TT-2014, as the International Structure of TTs. Finally, we refer to the countries in which a given TT appears in the first Δ*t* (i.e., 5 minutes) after the surge of a TT as *sources* since based on our model they cannot be followers of any other country for this TT.

### Heterogeneity in sharing behavior

The TTs of a country can be divided in those that stay local in the country (non-shared TTs) versus those that are shared. Furthermore, among the shared TTs, each country might act as a source if it is among the first set of countries in which the TT appeared, thus leading some following countries and contributing to the corresponding links in the network of Fig 2. The descriptive statistics on the amounts and ratios of shared TTs and on the frequency at which countries act as sources show two kinds of outliers (more details in S1 Text). First, some countries are practically isolated, for example Japan, Korea, and Turkey have more than 95% non-shared TTs, while the median is below 60%. Second, some countries have extremely central roles, such as the United States and Great Britain, sharing 67% and 50% more TTs and acting as source in two to three times more tweets than the third largest contributor (Canada).

The above observations of heterogeneity are consistent with a centralization pattern, which we empirically test to assess if countries pay a significantly higher attention to small groups of other countries. We measure the concentration associated to both leading and following activity at the individual country level, through the analysis of the distribution of in and out weights of the links in the international structure of TTs shown in Fig 2. We quantify the centralization of attention from and to a country *C*_*i*_ as the Gini coefficient of the weights of outgoing and incoming links, respectively. The Gini Coefficient measures inequality, varying from 0 (complete equality) to 1 (complete inequality). In our case, a high Gini coefficient is an indicator of centralization in either the attention a country pays or in the audience that follows the TTs of a country. As a result, for each country in each dataset, we produced two values of *leading* Gini and *following* Gini, each one measuring the local centralization around the country in either in-degree or out-degree.

To assess the statistical significance of the Gini coefficients, we compare the empirical estimates with two null models: an Erdos-Renyi random network (RN) [41] and a Susceptible-Infected-Recovered (SIR) process [42]. For the RN case, we produced ten random networks for each dataset, maintaining the amount of nodes, links, and total weight of the original graph. For the SIR case, we simulated ten times a simplified version of the process in the network [43] using the parameter *β* = 4/*N*, which fits the density of our empirical networks. For each simulation of RN and SIR we computed the leading and following Gini of each node in the same manner as we did for the empirical network.

**Fig 3:**
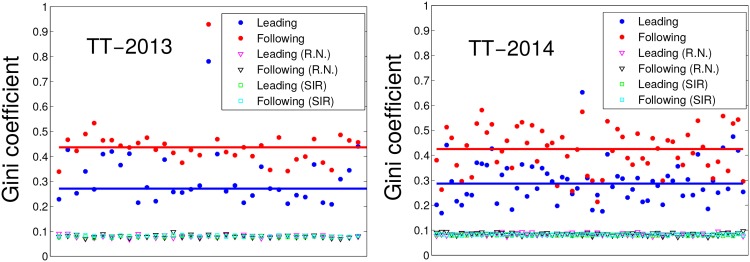
*Leading* and *Following* Gini coefficients. Each Gini coefficient is calculated for the networks of leading-following relations in each dataset and mean values for ten simulations of random networks and SIR processes. Horizontal lines show means over all countries.

Fig 3 shows the leading and following Gini coefficients for each of the countries in TT-2013 and TT-2014, and the averages of the corresponding nodes in the null models. The dispersion of the Gini values in the RN and SIR ensembles are very low, and cannot be appreciated at the scale of the picture. Horizontal lines show the means across nodes for each case, illustrating that the mean leading (following) Gini coefficient is 3.4 (5) times larger in the empirical graph than in the random graph ensemble for TT-2014 and 3.4 (5.5) for TT-2013. This shows an important level of centralization in both the attention attracted by countries from others and the attention countries dedicate to others. Moreover, the attention dedicated to others (following Gini) is more concentrated in fewer countries than the attention received (leading Gini). These results reveal that the network of leader-follower relationships does not only display heterogeneity in overall TTs production and source levels, but also is highly centralized at the local level in both attention and leadership aspects.

### Systemic and subjective biases of leader-follower relations

We empirically test the coexistence of subjective and systemic biases in the international coverage of TTs at the link level, using our TTs datasets. We follow a regression model similar to the ones previously applied to the analysis of mass media [16, 17], including geographic, demographic, economic, and cultural aspects of the involved countries.

Subjective biases would manifest as a stronger leader-follower relationship measured as the amount of *TT*_*xy*_ (i.e., TTs in which country *y* follows country *x*), when countries *x* and *y* have similar cultures. In this analysis, clearly the dependent variable is *TT*_*xy*_, which is hypothesized to depend on other factors of the countries involved. We measure the euclidean distance in cultural space as $C_{xy}=\sqrt{\sum_{v\in D}{\left(v_{x}-v_{y}\right)}^{2}}$, where *D* is the set of cultural dimensions in Hofstede’s model and *v*_*x*_ is the value of dimension *v* for country *x* as explained in the Data and Methods Section. If the propaganda model holds, systemic biases will increase the leadership of countries with high GDP towards countries with lower GDP, and thus the strength of leader-follower relationships will depend on the GDP difference *G*_*xy*_ = *GDP*_*x*_ − *GDP*_*y*_. Furthermore, systemic biases are hypothesized to reach beyond linguistic barriers, in comparison to subjective biases that should depend on shared languages. We quantify linguistic barriers through a binary variable *L*_*xy*_ that takes the value 1 if two countries share an official or national language, and 0 otherwise.

We test the existence of subjective and systemic biases controlling for the effect of timezones in our analysis. The fact that some countries start the day before others is subject to have an effect in leader-follower relationships, creating an additional bias in which leadership relationships tend to go from east to west. We include the time difference between the largest cities of each country *T*_*xy*_, subtracting the timezone of *y* from the timezone of *x*, and converting it to a 12 hour basis. In addition, demographic factors also have the potential to influence the international coverage of TTs, where migration patterns might change the relevance of TTs depending on the migration from leader to follower *m*_*xy*_ and from follower to leader *m*_*yx*_.

We fit a linear regression model with an interaction effect on language as:

$$\begin{array}{ccc} & & TT_{xy}=I+a_{e}m_{xy}+b_{e}m_{yx}+c_{e}T_{xy}+d_{e}G_{xy}+e_{e}C_{xy}\\ & & +L_{xy}(f+a_{i}m_{xy}+b_{i}m_{yx}+c_{i}T_{xy}+d_{i}G_{xy}+e_{i}C_{xy}) \end{array}$$ (1)

The above model is a combination of two linear models, one that holds for all pairs of countries regardless of their common languages, in which biases are *external* to language barriers and are quantified by *a*_*e*_, *b*_*e*_, *c*_*e*_, *d*_*e*_, *e*_*e*_. The second part of the model holds only when countries have some language in common (*L*_*xy*_ = 1), and represents the additional biases *internal* to language barriers *a*_*i*_, *b*_*i*_, *c*_*i*_, *d*_*i*_, *e*_*i*_. The intercept of the second part of the model (*f*) is a constant factor that is only present when *L*_*xy*_ = 1, and quantifies the *height* of the language barrier in comparison to the constant intercept *I*. Thus, *f* measures the bias in TTs that can be attributed only to the fact that two countries share a language. To be able to compare datasets, we renormalized all variables and filtered out countries without values in Hofstede’s dataset, analyzing a total of 870 pairs from TT-2013 and 1722 pairs from TT-2014.

Table 1 shows the regression results for the above model on the TT-2013 and TT-2014 datasets. The estimate of *d*_*e*_ is positive and significant for both datasets, showing the existence of a systemic bias that depends on the economy regardless of language barriers. In line with our theoretical argumentation, this suggests that TTs follow the gradient of wealth: TTs created in rich and big countries are more likely to appear in follower countries with less wealth and power. This bias has been also reported for the coverage of traditional news over mass media [44, 45]. We do not find evidence of an additional effect of this GDP bias internally to language communities (the estimates of *d*_*i*_ are much smaller than *d*_*e*_ or not significant), indicating that the systemic bias is indeed global and not influenced by language. The size effect of the GDP difference is relatively large, being the strongest effect present in the case when countries do not share a language. Migration effects do not provide consistent results, only showing some positive effect for the migration from follower to leader in TT-2014. We attribute this result to the larger coverage of TT-2014, which includes a broader range of countries and reveals the effect that immigrants in a host country make newsworthy the TTs from their home country.

**Table 1:** Regression results of TT model for TT-2013 and TT-2014.

	2013	2014
*I*	**−0.17** (0.04)***	**−0.13** (0.02)***
*a*_*e*_	0.21 (0.11)	0.16 (0.06)*
*b*_*e*_	0.00 (0.10)	**0.17** (0.06)**
*c*_*e*_	−0.04 (0.03)	−0.04 (0.02)
*d*_*e*_	**0.39** (0.05)***	**0.24** (0.03)***
*e*_*e*_	−0.03 (0.04)	−0.06 (0.02)*
*f*	**0.53** (0.07)***	**0.45** (0.05)***
*a*_*i*_	−0.14 (0.12)	−0.04 (0.07)
*b*_*i*_	0.15 (0.10)	0.03 (0.06)
*c*_*i*_	**0.20** (0.07)**	0.05 (0.05)
*d*_*i*_	−0.15 (0.07)*	−0.06 (0.05)
*e*_*i*_	**−0.40** (0.07)***	**−0.38** (0.05)***
R^2^	0.26	0.22
Adj. R^2^	0.25	0.22
Num. obs.	870	1722

***p < 0.001

**p < 0.01

*p < 0.05

The estimates of *e*_*e*_ and *e*_*i*_ reported in Table 1 show that cultural distance has a negative weight on the strength of leader-follower relationships, in particular within language barriers. The estimate of the external cultural bias is close to 0 for both datasets only significant in TT-2014, revealing an almost negligible influence across cultures that do not speak any common language. The estimate of the influence of language (*f*) is highly positive and significant, indicating that TTs are much more likely to follow leader-follower relationships of countries that share at least a language.

These results portray the international structure of TTs within and across language barriers. Across languages, money speaks and TTs follow GDP, also influenced to some extent by migration patterns. Within language communities, the bias of GDP is also present, but it is attenuated by stronger relationships between countries closer in cultural space. It is worth noticing that the role of time zones is not significant across languages and only significant within languages in the smaller TT-2013 dataset. The inclusion of *T*_*xy*_ in the model makes our estimates of the rest of biases robust to the effect of timezones. The positive estimate of *c*_*i*_ in TT-2013 portrays the phenomenon that TTs go from east to west when countries share a language, but this is not observable in the larger dataset.

We verify the significant and sizable values of *d*_*e*_, *f*, and *e*_*i*_ through permutation tests (see S1 Text), concluding that our results are robust to empirical correlations in the link structure of the network of TTs. Furthermore, the model has certain prediction power as measured through the adjusted *R* ^2^ values of 0.26 and 0.23, i.e., around 25% of the variance of the weight of leader-follower relationships can be explained by our model of systemic and subjective biases.

### The role of mass media in Twitter

Social media are not isolated from the content generated by mass media, which motivates our analysis of the interaction between both types of media. We apply our method to identify which TTs are associated to mass media content in 4 countries (US, GB, CA and ES), detecting which TTs are Internal (not reported in mass media) or External (also reported in mass media). In the following, we present an explorative study of the properties of TTs in relation to mass media, focusing on three aspects: i) the volume and properties of TTs that are reported in mass media, ii) the extend to which TTs detected in mass media are only local or travel along leader-follower relationships, and iii) the patterns of delay between the appearance of TTs and news reports.

**Fig 4:**
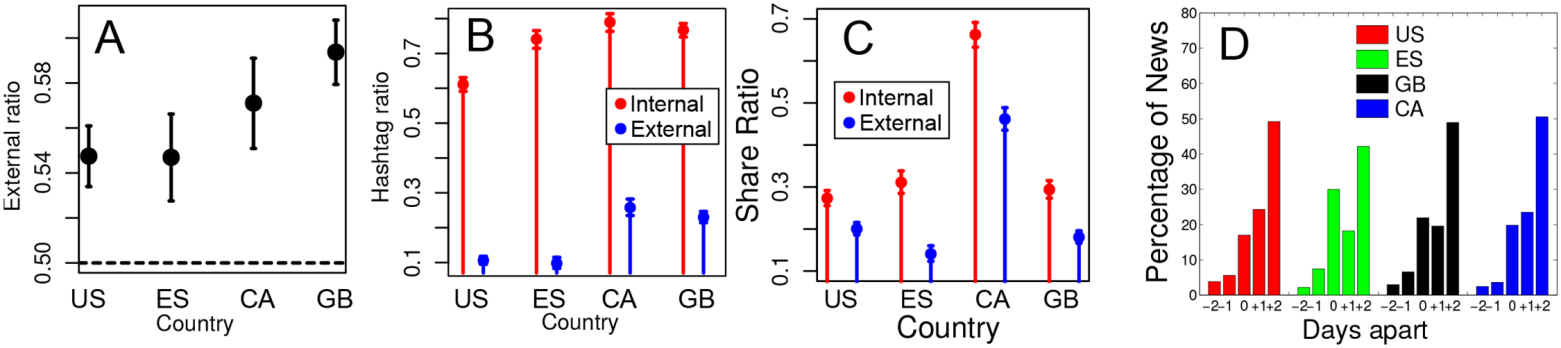
Properties of internal and external TTs. A) Ratio of External TTs; B) Ratio of External and Internal TTs that are hashtags; C) Conditional Probability of Internal or External TTs being shared; D) Percentage of news appearing in principal newspapers with some positive or negative delay with respect to Twitter TTs. All figures show the results of the analysis for US, ES, CA and GB. Error bars indicate the 95% confidence intervals from *χ* ^2^ tests.

Fig 4A depicts the percentage of External TTs (or External Ratio) for each one of the analyzed countries. This shows that Twitter is not isolated from mass media, since slightly more than half of the TTs overlap with news found through Google News. Despite this important interaction between Twitter and mass media, still roughly between 40–46% TTs are associated to content not reported in mass media. Our data shows that hashtaging is a clear differentiating factor between Internal and External TTs. Fig 4B presents the percentage of Internal and External TTs that are hashtags across the four studied countries. We observe that roughly 60–80% Internal TTs are hashtags whereas these values shrink to 10–25% for External TTs. This suggests that in order to become TT, without the back up of mass media channels, a centralized communication mechanism is required. The hashtaging functionality of Twitter provides such a mechanism, allowing both the collective discussion about the topic and the semiotic creation of a symbol to refer to it [46].

We have analyzed the TTs shared by each pair of the four considered countries. Note that if a TT is Internal in both countries, then it was shared internally within Twitter and it was not reported by traditional media in either country. Moreover, if a shared TT is External in any of the two countries, the sharing process may have occurred through either mass media or Twitter. Then, to be conservative, we consider that this TT was shared through external media in this case. Based on these considerations, Fig 4C shows the conditional probability that a TT classified as Internal (External) is shared between countries within Twitter (through mass media). We confirm that, for the four analyzed countries, TTs are more likely to be shared internally. Indeed, the probability of being shared internally is 50–100% proportionally higher than externally. In conclusion, the flow of TTs between countries is mainly formed by internal content generated in Twitter rather than news of interest to traditional mass media.

To conclude our analysis, we compare the surging dates of External TTs and their associated news in mass media to check in which communication venue overlapping news are (typically) reported earlier. Note that we are not considering individual tweets but TTs. It seems likely that an individual tweet may capture an event before it is reported in online traditional media. However, when that event becomes a TT it has the entity of a piece of news that has attracted the attention of a large number of people. We are interested in understanding if this happens before or after the event has been reported in mass media. Specifically, we have computed the difference in number of days between the appearance date of every external TT and its associated piece of news in online newspapers in the four considered countries. Note that the difference ranges between ± 2 days from the appearance date of the TT since this is the window time that we have configured to query the Google News service. Fig 4D shows the percentage of news that surged two days before, one day before, the same day, one day after and two days after in traditional media than in Twitter. More than 50% of the external TTs are reported earlier in Twitter than in the online edition of main newspapers whereas less than 10% of the news appeared earlier in those newspapers, and the rest appeared the same day in both channels.

## Discussion

We showed how news in social media manifest through Local TTs in Twitter, analyzing two alternative large-scale datasets of TTs in different countries. We validated our analysis of the leader-follower relationships between countries testing the hypothesis of priority processes in queue systems [34, 35], finding a power-law distribution of delay times between the appearance of TTs. This finding conveys knowledge about the dynamics of how TTs travel across countries, in an analogous manner as how power-law degree distributions reveal dynamics of preferential attachment or edge copying mechanisms [47]. Applying the statistical physics of priority processes has potential applications to the analysis of communication dynamics in other online communities, from dialogues to collective reactions.

Leader-follower relationships among countries reveal patterns of heterogeneity in sharing activity as well as concentration of attention, allowing us to test hypotheses inspired in mass media about the role of economic and social factors [6, 14, 15]. We found that content in social media, similarly to mass media, follows the gradient of wealth from rich to poor countries. Our combination of data about TTs with Hofstede’s quantification of culture [10] contributes to the wider scientific field of online ethnography [48]. Our results resonate with works about the online manifestation of cultural traits [12, 49, 50], unemployment [51], and economic inequality [52]. Similarly as how our analysis of TTs showed how information in social media can cross international borders, the analysis of digital traces has the potential to explain many other individual and collective aspects of human behavior.

To analyze the role of mass media in reported TTs, we designed a method to match TTs to news in mass media close to the TT appearance. Using this method, we found that internal TTs are much more likely to manifest around a hashtag, which serves as a symbol to centralize communication in the absence of important mass media channels. This method also allowed us to statistically control for mass media in how TTs are shared across countries, revealing that external TTs are less likely to cross country borders in Twitter than those TTs that were not considered newsworthy by mass media. Further applications of this tool have the potential to enhance the analysis of dynamic collective response patterns in Twitter [46], allowing the measurement of reach and social interaction around news channels.

In this context, Kwak et al. [53] analyzed the overlapping between TTs and mass media news in 2009. First, the authors confirm the condition of breaking news of TTs. Furthermore, they compare news coverage by World Wide Twitter TTs with Hot Topics from Google Trends and headlines of CNN news concluding that CNN was ahead in reporting. Our explorative analysis suggests that this situation has been reversed in the last years, and now Twitter seems to be ahead mass media, at least for the part in which TTs overlap with traditional news channels. Roughly half of TTs overlap with news reported by mass media indicating an important interaction between both venues. Despite this high interaction and the common systemic biases in the international coverage of mass media news and TTs, the major fraction of TTs shared across countries in Twitter correspond to events not reported by major mass media.

In summary, our work shows how content in social media can break international borders through Twitter TTs, revealing that Twitter is used as an alternative communication channel with respect to mass media. On the other hand, we found significant biases with respect to economic, demographic, and cultural factors. This portrays Twitter as a mixed and multipurpose community, in which content can flow without constraints, but also in which mass media have a strong influence and in which economic and cultural factors bias the flow of content.

## Supporting Information

S1 Text Quantifying the Economic and Cultural Biases of Social Media through Trending Topics. Details about methods, descriptive statistics, and analysis.
(PDF)
