# Supplementary material for: Quantifying the Economic and Cultural Biases of Social Media through Trending Topics
Source: PLoS One. 2015 Jul 31;10(7):e0134407. doi: 10.1371/journal.pone.0134407 (PMC4521871; doi:10.1371/journal.pone.0134407)
Supplement: S1 Text — Details about methods, descriptive statistics, and analysis. (PDF) [file pone.0134407.s001.pdf]

Quantifying the Economic and Cultural Biases of Social Media through Trending Topics – Supporting Information

Juan Miguel Carrascosa[1], Ruben Cuevas[1], Roberto Gonzalez[2], Arturo Azcorra[1,3], David Garcia[4,*]
[1] Univ. Carlos III de Madrid, Madrid, Spain
[2] NEC Laboratories, City, State, Country
[3] Institute IMDEA Networks, City, State, Country
[4]Chair of Systems Design, ETH Zurich, Zurich, Switzerland
* jcarrasc@it.uc3m.es, rcuevas@it.uc3m.es, roberto.gonzalez@neclab.eu, azcorra@it.uc3m.es, dgarcia@ethz.ch

June 26, 2015

1 Method for mass media TT detection

We have implemented a method that uses the Google News services to infer if a TT appearing on date d has been reported in the mass media of a specific country in a time window of $d \pm N$ days. For a given TT, our method is divided into a *pre-processing phase* to translate the TT into an appropriate format to query the Google News service, and a *search phase* in which the Google News service seeks for news including the words forming our TT.

The **pre-processing** phase is divided into two steps. First, (when required) we transform the TT in a set of meaningful words. For instance a TT "#BarackObamaInNewYork" would be transformed into "Barack Obama In New York" (the # is removed and the words forming the TT are properly separated). Second, we filter all the words contained in the lexicon of the top 1000 most frequent words of the language of the country, constructing a set of terms to query without common words that can easily produce false positives. If all the words in the TT are included in the most frequent lexicon, that TT is automatically filtered out in this phase.

In the **search** phase we access the Google News service with the following information: (*i*) we query for the keyword(s) produced by the pre-processing phase; (*ii*) a filter on a specific media outlet depending on the country as explained below; and (*iii*) a time window to perform the search. Specifically, we configure the search of Google News to only consider news pieces that were produced within the designated period, and to look for the keyword(s) in headline and the body of the articles. Therefore, if the result of the *search* phase includes at least one piece of news, we classify the associated TT as External (i.e., reported in Twitter and mass media), and categorize the TT as Internal (i.e., exclusively reported in Twitter) otherwise. Moreover, we also record the earliest date of the news returned by Google News for each External TT. We

acknowledge that the proposed methodology may produce some miss-classifications. For instance, a TT #BarackObamaInNewYork would be classified as external if a news piece with a headline *'Barack Obama's administration passes a new Education bill'* appears in the considered time interval ($d \pm N$ days). Despite both events are different, both of them present the common words 'Barack Obama'. To the best of the authors knowledge there is not an available ground truth dataset that would allow to validate our methodology. In the absence of such ground truth, we provide an initial validation of our method through a qualitative pull-out **?** using a random set of 1000 TTs from Spain. An independent rater (a student not taking any other part on this research) was instructed to indicate whether the semantic content of the word(s) of a TT describes current news or not (e.g., in the case of including exclusively very common words). Therefore, those TTs classified by the rater as not news should be filtered out by our algorithm in the pre-processing phase. The result of this pull-out is that 96% of the considered TTs are equally classified by the rater and our method. A χ^2 test on this result shows that the 95% confidence interval of the accuracy of our method is $[0.945, 0.971]$, and thus it is safe to assume that our method accurately filters out those not newsworthy TTs during the pre-processing phase.

We have applied the described method to the TTs of four countries (US, ES, CA and GB) collected over a period of time of 1 month between April and May, 2014. This includes 5297, 2598, 2385 and 4598 TTs from US, ES, CA and GB respectively. When accessing the Google News service, we focus on the online versions of three large news papers of each country[1]. Note that considering all indexed media by Google News in a country would generate significant noise (e.g., blogs that report the list of Trending Topics) in our results. Instead, the online version of main newspapers provide a broad coverage of different type of news (breaking news, politics, sports, society, science, etc) and at the same time are quite dynamic venues that rapidly report any major event happening. Therefore, they are an appropriate venue to identify the most relevant news in a country. Finally, we filtered news reporting for a time window of 5 days around the appearance of the TT, and repeated the same detection technique for a window of 7 days, obtaining very similar results due to the breaking nature of news.

[1] New York Times, Washington Post and USA Today for US. El Pais, El Mundo and ABC for ES. The Globe And Mail, National Post and Vancouver Sun for CA. Telegraph.co.uk, The Guardian and The Independent for GB.

2 Descriptive statistics of TT

For each country in our TT-2013 and TT-2014 datasets, we computed the total number of associated TTs as well as its break down into those that remain local within the country (i.e., are not shared with other countries) and those shared with at least another country. These metrics allow us to understand how much individual countries share TTs internationally. In addition, a critical aspect to define the importance of a country is its capacity to generate TTs that are afterwards consumed by others. According to our leader-follower model, this happens when a country is a source for a TT that is afterwards consumed by others. To characterize the bias of countries towards generating or consuming TTs we compute the *Source Ratio* (SR) as the ratio between the number of TTs in which the country acts as a source and the number of TTs in which it is not a source but a consumer.

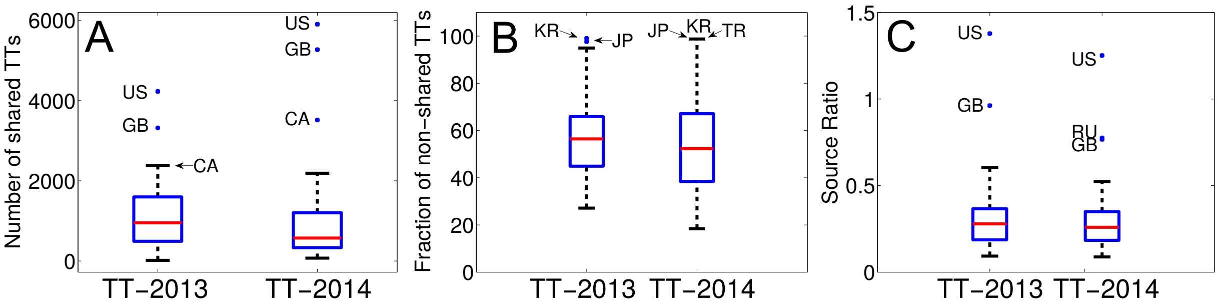

Figure 1: **Heterogeneity in TT sharing behavior**. Boxplots of the number of shared TTs (A), the fraction of non-shared TTs (B) and the source ratio (C) for the countries in both TT-2013 and TT-2014 datasets. Red lines show median, boxes 25 and 75 percentiles, bars represent 1.5 times the interquartile range, and outliers are marked.

Figure 1 presents the distribution of the following three metrics across the countries represented in TT-2013 and TT-2014 in the form of a boxplot: (*i*) total number of shared TTs, (*ii*) fraction of non-shared TTs and (*iii*) SR. Overall, in both datasets we observe a significant heterogeneity across the three metrics. For instance, in the case of TT-2014, the interquartile range and the max-min difference for each metric are: (*i*) 871 and 5832, for the number of shared TTs, (*ii*) 28.69% and 80.37%, for the fraction of non-shared TTs, and (*iii*) 0.17 and 1.25, for the SR. Note that, as Figure 1 shows, the results are similar for TT-2013.

3 Inter-event time distribution analysis

year	N	xmin	α	KS	λ	R	p-value
2013	152938	300	$1.000047(10^{-6})$	0.066	$7.9 * 10{-}6$	198.2	0.0
2014	279247	300	$1.0041(10^{-5})$	0.076	$9 * 10{-}6$	293.03	0.0

Table 1: **Summary of inter-event distribution fits.** Statistics of power law and exponential fits for inter-event distributtions in TT.

4 Permutation tests of TT regression model

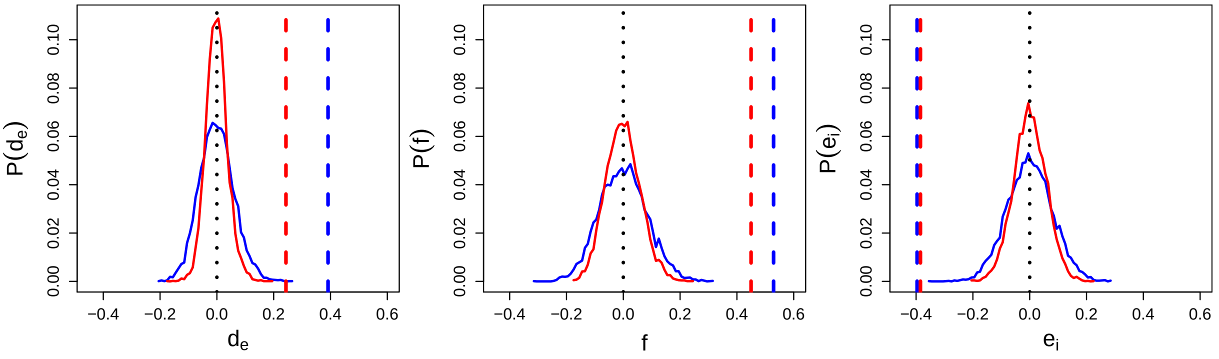

Figure 2: **Permutation tests of regression results**. Distributions of estimates of d_e, f, and e_i for 10.000 permutations of TT_{xy} for TT-2013 (blue) and TT-2014 (red). Vertical lines show the point estimates of OLS regression.
